# Building International Capacity for Citizen Scientist Engagement in Mosquito Surveillance and Mitigation: The GLOBE Program’s GLOBE Observer Mosquito Habitat Mapper

**DOI:** 10.3390/insects13070624

**Published:** 2022-07-13

**Authors:** Russanne D. Low, Theresa G. Schwerin, Rebecca A. Boger, Cassie Soeffing, Peder V. Nelson, Dan Bartlett, Prachi Ingle, Matteo Kimura, Andrew Clark

**Affiliations:** 1 Institute for Global Environmental Strategies, Arlington, VA 22202, USA; theresa_schwerin@strategies.org (T.G.S.); cassie_soeffing@strategies.org (C.S.); andrew_clark@strategies.org (A.C.); 2 The Department of Earth and Environmental Sciences, Brooklyn College, Brooklyn, NY 11210, USA; rboger@brooklyn.cuny.edu; 3 College of Earth, Ocean, and Atmospheric Science, Oregon State University, Corvallis, OR 97331, USA; peder.nelson@oregonstate.edu; 4 Northwest Mosquito Abatement District, Wheeling, IL 60090, USA; dbartlett@nwmadil.com; 5 Department of Computer Science, University of Texas at Austin, Austin, TX 78705, USA; prachi.ingle@utexas.edu; 6 Independence High School, Frisco, TX 75035, USA; mateus.sakata@gmail.com

**Keywords:** citizen science, mosquito, smartphone, mobile app, vector-borne disease, vector surveillance, mitigation, community engagement, open data

## Abstract

**Simple Summary:**

The GLOBE Program’s GLOBE Observer Mosquito Habitat Mapper is a free citizen science data collection tool that can be downloaded onto smartphones. The Mosquito Habitat Mapper encourages individuals to participate in locating and removing mosquito breeding habitats from use. An easy-to-use graphic interface enables users to report and describe mosquito breeding habitats, places with standing water where immature mosquitoes grow and develop. Citizen scientists are asked to determine if they see immature mosquitoes and if they wish to count and identify any mosquito larvae they see. In the last task, the user is asked to dump out or cover the standing water source, eliminating its use as a breeding habitat. In this way, the GLOBE Observer mobile app also supports the actions of individuals protecting their communities from mosquito-borne disease. In addition, all data reported by citizen scientists are publicly available. Scientists are accessing this data for a variety of research uses, including the development of automated techniques to recognize larvae and mosquito breeding sites from digital images. Since 2017, more than 32,000 Mosquito Habitat Mapper observations have been submitted by citizen scientists in 84 countries.

**Abstract:**

The GLOBE Program’s GLOBE Observer Mosquito Habitat Mapper is a no-cost citizen scientist data collection tool compatible with Android and iOS devices. Available in 14 languages and 126 countries, it supports mosquito vector surveillance, mitigation, and education by interested individuals and as part of participatory community surveillance programs. For low-resource communities where mosquito control services are inadequate, the Mosquito Habitat Mapper supports local health action, empowerment, and environmental justice. The tangible benefits to human health supported by the Mosquito Habitat Mapper have encouraged its wide adoption, with more than 32,000 observations submitted from 84 countries. The Mosquito Habitat Mapper surveillance and data collection tool is complemented by an open database, a map visualization interface, data processing and analysis tools, and a supporting education and outreach campaign. The mobile app tool and associated research and education assets can be rapidly deployed in the event of a pandemic or local disease outbreak, contributing to global readiness and resilience in the face of mosquito-borne disease. Here, we describe the app, the Mosquito Habitat Mapper information system, examples of Mosquito Habitat Mapper deployment in scientific research, and the outreach campaign that supports volunteer training and STEM education of students worldwide.

## 1. Introduction

Reducing the health risks associated with mosquito-borne disease is a critical humanitarian challenge. In virtually every country battling endemic mosquito-borne diseases, the constant need for mosquito surveillance and mitigation dwarfs the available funds and professional personnel that can be allocated to these tasks. Simply put, the scale of the problem of mosquito-borne disease requires an increased commitment to supporting crowdsourcing surveillance and mitigation efforts.

Mosquito-borne disease outbreaks are a collateral threat accompanying contemporary climate change. Vector-borne diseases once limited to tropical and subtropical zones are now appearing in temperate regions [1,2,3]. An increased frequency of extreme weather events, rapid urbanization, accelerated human mobility, and international travel and trade expand the risk of human exposure to mosquito disease vectors [4]. As a result, 80% of the world’s population is now at risk of mosquito-borne disease, and more than 700,000 people die from these diseases every year [5].

Adequate mosquito control is an essential defense against vector-borne disease. However, there is a nearly universal need for increased mosquito surveillance and habitat mitigation in communities worldwide. In most municipalities, the costs associated with highly effective vector management programs are prohibitive, while remote and rural communities often have no access to mosquito surveillance or mitigation services. In many regions, already insufficient mosquito control measures were further constrained by the reallocation of public health personnel and resources to address the immediate threat of the COVID-19 pandemic [6,7,8,9].

To offset these challenges, community-based vector monitoring programs are springing up worldwide [10]. Broad access to mobile devices has enabled the activation of community volunteers as a cost-effective solution to improve the spatial and temporal coverage of mosquito surveillance and mitigation in communities, while simultaneously improving public awareness of vector-borne disease transmission. This is particularly important in rural and remote communities with no active mosquito monitoring programs [11]. Volunteers share their experiences and knowledge, and improve local awareness about the behavioral practices that can reduce mosquito-borne disease risk [12].

Citizen science is a form of research that welcomes public participation in identifying research questions, collecting and analyzing data, interpreting results, and developing technologies and applications. Broadening public engagement with science supports new discoveries and solutions to some of the world’s most pressing problems. In the last decade, participatory research projects have grown in popularity, with millions of people volunteering on projects every year [13,14]. The potential value of spatiotemporal data reported by non-specialist volunteers using personal mobile devices has been recognized by environmental scientists and public health researchers [15,16,17,18].

The last decade has seen the development of numerous mosquito surveillance projects, and several citizen science strategies are tailored to address health challenges in specific geographic areas of interest (Table 1). The projects vary not only in spatial granularity (e.g., local, regional, national, international), but also in the categories of data collected (e.g., location of adult mosquitoes or standing water breeding sites, reports of source reduction, and target species).

Community engagement strategies and reporting tools also vary. Citizen scientists may be asked to mail in specimens, or upload data using a web platform or mobile app. A few projects seek to engage youths in surveillance [20,25,26,27,28,33,34,35,48], and some specifically mention a commitment to open data access [25,26,27,32,33,38,41].

The value of including community residents in participatory surveillance is obvious in the case of *Aedes aegypti* and *Aedes albopictus*, two invasive species whose ranges are expanding rapidly worldwide: 13 of the 36 citizen science projects described above have targeted surveillance of these two species. Spurred by a warming climate and economic globalization, the range expansion of *Ae. aegypti* and *Ae. albopictus* container-breeding mosquito disease vectors pose formidable challenges to vector control agencies. These species habitually oviposit in ephemeral puddles of water that are often cryptic, ubiquitous, and difficult to access in and around home, causing vector surveillance and mitigation to be increasingly labor-intensive and costly.

Finally, the position, numbers, and kinds of water containers found in domestic landscapes are modified by residents regularly, confounding vector control predictions of where and when these species may be found [54]. The use of containers as oviposition sites also enables these species to evade expectable seasonally linked population fluctuations, as immature mosquitoes can persist throughout dry periods in these microhabitats [55].

Examined together, these projects serve as an informative international field experiment testing and documenting the effectiveness of various crowdsourced mosquito surveillance strategies in diverse cultural contexts. As a contribution to this effort, we present the citizen science tool, GLOBE Observer Mosquito Habitat Mapper, and describe features of the outreach program that supports its adoption and use. Referring to Table 1, the Mosquito Habitat Mapper shares many features with other projects, including leveraging smartphone technology to collect photo voucher data, supporting local mosquito surveillance and mitigation, and educating the public about the health threat of mosquito disease vectors.

The Mosquito Habitat Mapper also presents some unique citizen science innovations related to both NASA science and NASA’s commitment to open data and public STEM (science, technology, engineering, and math) outreach and education. These features include a global network of citizen scientists contributing data to a publicly available database for research, a flexible data collection protocol that supports its application to a wide variety of research questions, and a deliberate youth engagement strategy that includes working with students, teachers, and informal science educators.

**Figure 1:**
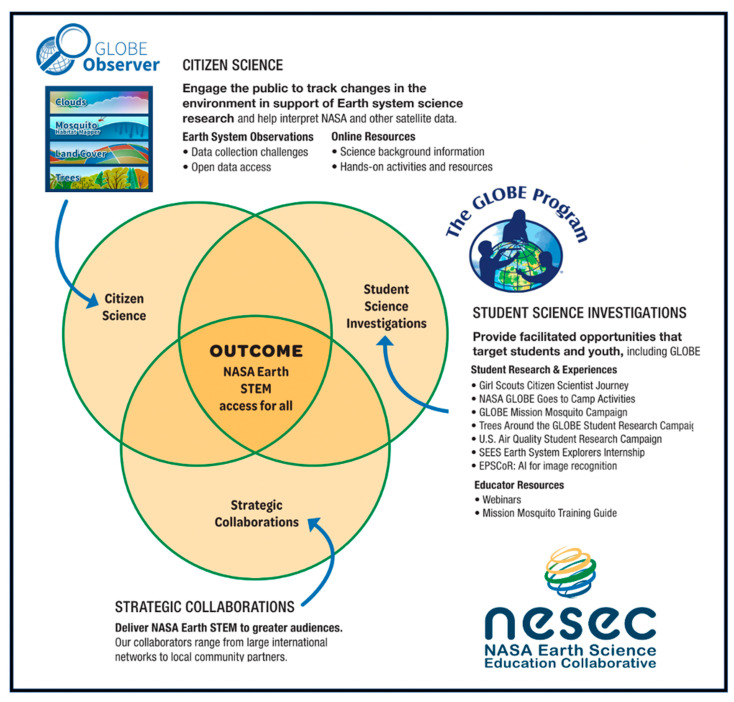
GLOBE Mosquito Habitat Mapper is a strategic active engagement component of the integrated education and outreach effort led by the NASA Earth Science Education Collaborative [56].

The Mosquito Habitat Mapper is distinguished from many other citizen science mosquito surveillance projects by its supporting pedagogic infrastructure and emphasis on education. The app tool is supported by the NASA Earth Science Education Collaborative (NESEC). NESEC is an initiative led by the Institute for Global Environmental Strategies (IGES), in close partnership with the Earth science programs at NASA’s Goddard Space Flight Center, Jet Propulsion Laboratory and Langley Research Center (Figure 1). As an embedded programmatic element of a STEM education initiative, the Mosquito Habitat Mapper’s potential contributions to both scientific data and Earth system education are valued equally.

**Figure 2:**
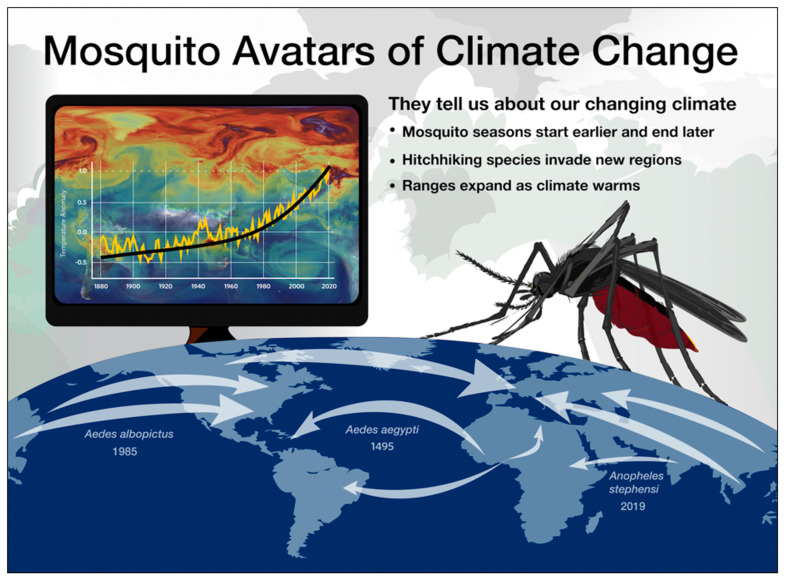
Infographic used by GLOBE Mosquito Habitat Mapper science outreach team in public climate change education. Image credit: Jenn Paul Glaser and Russanne Low.

In addition, the Mosquito Habitat actively engages citizen scientists in NASA’s Earth- observing missions. Citizen scientists using the tool collect ground-based data that complement remotely sensed environmental data obtained from satellite platforms [57]. These Earth-observing satellites have documented changes in the Earth’s climate. While contemporary changes in temperature are barely perceptible to most people, immature mosquitoes respond sensitively to seasonal, episodic, and directional changes in precipitation, temperature, and humidity. Warming temperatures over the past 40 years have resulted in a longer mosquito season; since 1980, more than 100 cities in the US have experienced an increase in their mosquito season by at least one week [58]. Invasive mosquitoes, once restricted to the tropics and subtropics, are now expanding poleward. Effective climate change communication has been a stubborn challenge for the scientific community [59], and we have seen the usefulness of the Mosquito Habitat Mapper as a climate change education and communication tool in social media and workshops (Figure 2).

In this article, we present the Mosquito Habitat Mapper tool and the data and outreach features that support its adoption by researchers, operational mosquito control agencies, communities, and the science-engaged public. In the Materials and Methods section, we begin by describing how the Mosquito Habitat Mapper supports the student research and science education goals of The Global Learning and Observations to Benefit the Environment Program (GLOBE). Next, we describe the data collection and access infrastructure. We discuss how these features promote data quality and challenges we have identified where user error can potentially bias data outcomes. In the Results section, we present examples demonstrating how the Mosquito Habitat Mapper supports scientific research and describe how the associated educational and outreach programs stimulate data collection. In the conclusion, we address data density challenges and provide a discussion of the importance of a broadly accessible citizen science mosquito surveillance tool that can be deployed in rapid response to mosquito-borne disease threats.

## 2. Materials and Methods

Whether data are collected by professional scientists or citizen scientists, methods need to be established and documented to ensure data quality and usability. This section discusses the design of the GLOBE Mosquito Habitat Mapper app and database as well as data quality features and challenges, accessibility, and interoperability.

### 2.1. The GLOBE Program

GLOBE is an international science and education program providing students and the public with the opportunity to contribute meaningfully to our understanding of the Earth system through their participation in environmental data collection and analysis [60,61,62,63]. For over 25 years, GLOBE has convened a community of practice, connecting scientists, teachers, and students in local data collection, documenting environmental conditions, and monitoring the changes taking place within the Earth system. Students explore questions related to the Earth’s hydrosphere, lithosphere, atmosphere, and biosphere by applying rigorous, standardized scientific data collection and analysis procedures designed by disciplinary scientists. All data submitted by program participants are archived in the GLOBE database. GLOBE currently operates in 126 member countries, and the GLOBE community has submitted more than 200 million observations since 1995.

**Figure 3:**
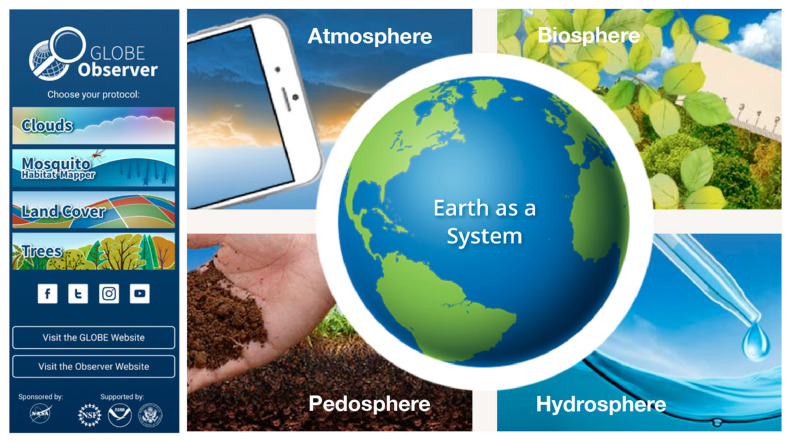
GLOBE Observer Mobile Application, supporting scientific investigation of the Earth’s system: Clouds (atmosphere), Mosquito Habitat Mapper (hydrosphere), Land Cover (pedosphere and biosphere), and Trees (biosphere). Modified from original: The GLOBE Program.

The GLOBE Program is NASA’s largest and longest running citizen science program monitoring the Earth. With the release of the GLOBE Observer mobile app in 2016, GLOBE expanded its participation outside the school setting to include citizen scientists of all ages. By leveraging the popularity and technological capabilities of handheld devices, the GLOBE Observer app broadens public participation in NASA science [57]. Earth observations can be reported using one or more of the four in-app data collection tools: Clouds, Land Cover, Trees, and the Mosquito Habitat Mapper (Figure 3). Citizen scientists are encouraged to submit spatially and temporally coincident observations using two or more tools because it allows them to observe and document some of the complex Earth system interactions that are responsible for the changes currently taking place on our planet.

The GLOBE Observer app connects users and their field observations to data collected by NASA’s satellite-based Earth-observing mission. The Clouds and Land Cover tools have built-in features that allow users to compare their ground-based observations to remotely sensed data obtained by satellites. The overpass notification feature built into the Clouds tool alerts users when to take an observation, and they receive a report that allows them to readily compare their own ground-based cloud observations to satellite images retrieved from a concurrent overpass [64]. Using the data visualization feature on the Land Cover tool, citizen scientists can confirm if their own land cover observations correspond to classifications obtained using the Moderate Resolution Imaging Spectroradiometer (MODIS) sensors aboard the Terra and Aqua satellites. The satellite data comparisons inform users of the importance of their in-situ ground reference data to scientists who analyze and interpret digital data derived by sensors on satellite platforms.

### 2.2. GLOBE Mosquito Habitat Mapper

Released in 2017, The Mosquito Habitat Mapper enables citizen scientists to report in situ ground-based observations of mosquito breeding sites. Citizen scientists using the Mosquito Habitat Mapper tool contribute to data gaps in NASA science, while participating in the community-scale mosquito larval surveillance and mitigation actions recommended by international health agencies [65] to reduce local mosquito-borne disease risk [66,67]. For low-resource communities where mosquito control services are inadequate, the Mosquito Habitat Mapper can serve as a tool of local empowerment and environmental health justice. The tangible benefits to human health supported by the Mosquito Habitat Mapper have encouraged its wide adoption, with more than 32,000 observations submitted from 84 countries.

### 2.3. Data Collection

The Mosquito Habitat Mapper was designed to accommodate citizen scientists who wish to participate at both casual and dedicated levels of engagement. Users are prompted to conduct a series of guided data collection tasks, including answering yes/no questions, uploading photos, sampling standing water and counting larvae, and identifying mosquito specimens, aided by a built-in pictorial taxonomic key.

**Figure 4:**
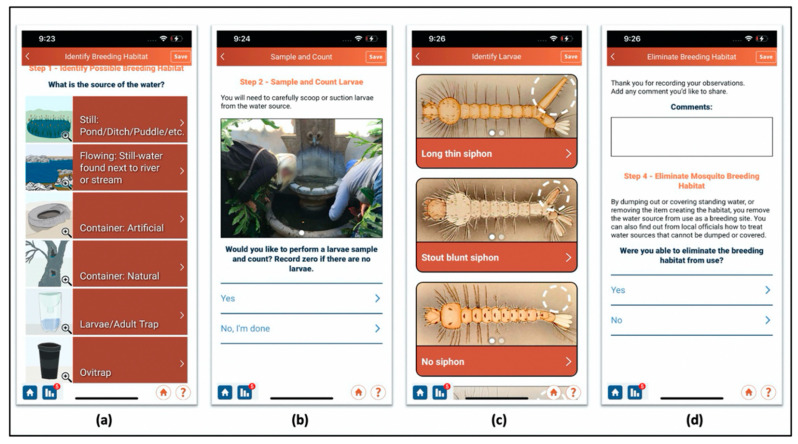
Screen captures from each of the four data collection steps. (**a**) Larval habitat documentation, (**b**) sample and count larvae, (**c**) larva identification, (**d**) source reduction (larval habitat mitigation). Source: The GLOBE Program.

Data collection proceeds stepwise through user interaction with a graphic interface. Consecutive steps increase in complexity and allow the citizen scientist to choose whether to attempt the more complex observations. This interactive user design feature opens citizen science participation to individuals across a broad range of ages (13+) and skill levels, from novice to professional scientist. Voucher photos are collected at each step to support data validation. The steps are identified in Figure 4 and described below.

#### 2.3.1. Step 1: Larval Habitat Documentation (a)

Spatial and temporal coordinates. The date, time, and geolocation of the habitat observation is automatically recorded from the device’s operating system and internal Global Positioning System (GPS) receiver. The GPS receiver simultaneously populates a map interface on the app, enabling the user to verify the location. GPS-enabled smartphones in an open sky environment typically display accuracy of ±5 m with accuracy decreasing with obscured sky conditions (buildings, bridges, trees, etc.) [68]. Depending on the device and conditions, locations with error estimates between 7–13 m can be expected in urban settings [69]. To obtain the highest positional accuracy possible, the user needs to wait a few moments for the mobile device’s GPS receiver to obtain a fix on the satellites and hit the “refresh” button several times until the desired accuracy is achieved. Latitude and longitude data can also be entered manually, either using data from an external GPS receiver, or by identifying the location on an auto-generated map interface.

Describing the larval habitat. The app presents images of 32 different categories representing natural standing water environments, natural containers (such as tree holes), artificial containers (such as tires or cisterns), or experimental habitats (such as larvae traps or ovitraps). “Other” is also an option. Users select the habitat type they observe and are instructed to photo-document the habitat and its immediate surroundings. Up to nine digital images can be captured at this step. At the end of this step, the user is prompted to indicate whether they can see mosquito larvae in the water (yes/no) and if they want to continue making observations.

#### 2.3.2. Step 2: Sample and Count Larvae (b)

Users are asked if they want to count and photo document larvae found in the water sample. At this point users can select to exit the tool or continue with data collection. To conduct this step, the user will need to use a 300 mL cup, dipper, or bulb syringe to extract a water sample. Larvae and pupae are tallied separately, and the presence/absence of eggs or nearby adults are recorded. To accommodate a variety of research projects, there is a text box where the user can enter field notes pertinent to their investigation, such as shade, water clarity, or the presence of aquatic vegetation.

If larvae are observed in this step, users are prompted to photo document one or more representative specimens, using the camera’s zoom feature on their mobile device. Interested citizen scientists are encouraged to obtain an inexpensive (less than USD 10) 60–100× clip-on macro lens, to obtain digital images of a sufficient resolution to enable taxon identification. Users are prompted to take several photos of their specimen(s), including one that captures the whole body, as well as close-up images of the diagnostic features located on the anterior and posterior ends of the specimen. Up to nine mosquito images can be saved during a single observation. Voucher photos of the larvae are uploaded at their original resolution to the GLOBE database.

#### 2.3.3. Step 3: Taxon Identification (c)

Users are asked if they want to proceed with taxonomic identification of their larval specimen. The citizen scientist’s own mosquito larvae photos are displayed in the user interface at each identification step for convenient comparison with diagnostic images in the key. After determining that the specimen is a mosquito larva, the citizen scientist uses the in-app pictorial dichotomous key to determine if their specimen belongs to one of three medically important genera found worldwide: *Aedes*, *Culex*, and *Anopheles* [5]. The key also distinguishes whether an *Aedes* specimen can be assigned to the species *Ae. aegypti* or *Ae. albopictus,* two widespread invasive mosquitoes that are also competent vectors for arboviruses that cause disease in humans. A text box is provided for users who wish to report taxa not included in the in-app taxonomic key.

#### 2.3.4. Step 4: Source Reduction (d)

Users respond to the prompt: “Were you able to eliminate the breeding site from use?”. If the citizen scientist can dump out or cover a container, remove trash, or otherwise mitigate the breeding site, they can select “yes”. This step reinforces the practice of source reduction, an important mitigation practice known to have a measurable impact on the transmission of mosquito-borne diseases within a community [66,67].

**Figure 5:**
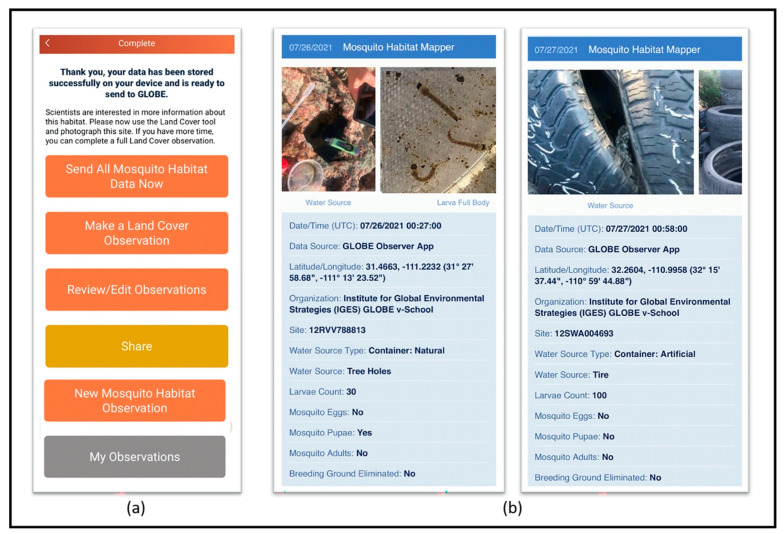
(**a**) Screenshot of the final frame of the Mosquito Habitat Mapper. (**b**) Example data records accessible via the GLOBE Observer mobile application interface. Source: The GLOBE Program.

When the citizen scientist has completed all the observations they wish to make in that session, the last frame presents several options. The choice, “Make a Land Cover observation”, connects the user to the GLOBE Land Cover tool on the same platform and enables the collection of land cover photos surrounding the mosquito larval habitat. Other choices include make a new observation, review the current observation, consult a list of past observations, share observations to social media (Facebook, Twitter, Pinterest), or upload data to the GLOBE database (Figure 5).

The app can be used offline. All observations are stored in the app until data services are accessible.

### 2.4. Data Access

A citizen scientist’s own observations can be examined and reviewed at any time in the app by selecting “My Observations” (see Figure 5b). Submitted observations are unloaded to the GLOBE database and are publicly accessible through the GLOBE Data Information System (DIS) [70]. Data can be accessed using a variety of tools.

**Figure 6:**
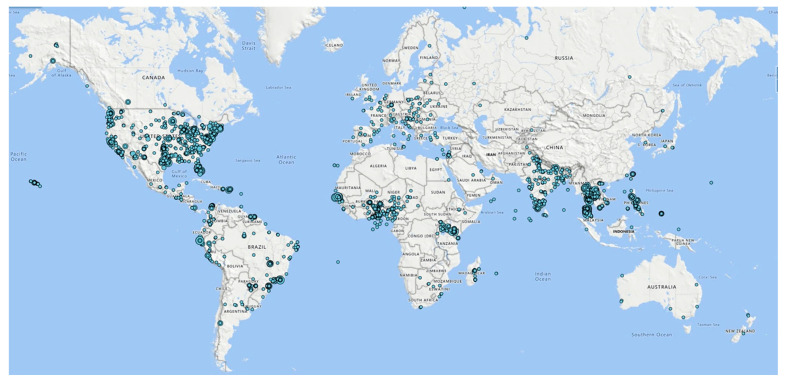
Locations of submitted Mosquito Habitat Mapper citizen scientist observations (29 May 2017–12 March 2022). (*n* = 32,894). Source: GLOBE Visualization System. Larger circles indicate a greater density of mosquito habitat observations in that location.

The GLOBE Visualization System displays a map interface with selected data for a specific date or range of dates (Figure 6). It also enables the diachronic examination of data by generating scientific time series visualizations. The GLOBE Visualization System can download spatial data as Keyhole Markup Language (KML) files for use in GIS applications.

GLOBE’s Advanced Data Access System (ADAT) can retrieve multiple categories of GLOBE data simultaneously, filtering by country, team, school, and geolocation. These data are returned in a commaseparated value (CSV) file. Metadata, including variables, units, and definitions are documented in the GLOBE Data User Guide [71]. An Application Programming Interface (API) is also available and returns data as a CSV file. Jupyter notebooks support the extraction of filtered data from the database.

In addition to the comprehensive GLOBE data access available through the GLOBE Data Information Services, IGES has created the Earth System Data Exploration Portal (beta), an integrated cloud platform for geospatial data analysis, community engagement, and collaboration [72]. Built on ESRI’s ArcGIS Online Open Data Hub framework, the portal offers a wide range of capabilities to support citizen scientist and student access of GLOBE Observer data. Filtered datasets are accessible in several geospatial formats, and the platform offers cloud-based geospatial analysis, web map creation, and cloud-deployed Python notebooks and image analysis workflows. Data for GLOBE Observer protocols are pulled at least every 24 h via an API call to the GLOBE API endpoint, resulting in regularly updated feature layers hosted on the portal.

In addition to these capabilities, the portal hosts curated datasets. These include annual datasets for GLOBE Mosquito Habitat Mapper, Land Cover and Clouds, outcomes of periodic data challenges, and a repository of datasets used in publications. The portal also hosts a library of community-created code products, such as a publicly available Python package and user interface, which expedite post-processing and quality assurance of GLOBE data [73]. Together, the raw data, metadata, and tools provided through the Earth System Data Exploration Portal enable researchers to evaluate the suitability of GLOBE Mosquito Habitat Mapper and Land Cover data for their specific scientific application.

### 2.5. Information Quality

Publicly collected data create challenges for analysis and require the development of robust data standards, practices, and procedures that assist scientists in determining the fitness for use of crowd-sourced data sets in research applications [13]. In the biological and environmental sciences, many citizen science programs accept data collected “anywhere and anytime”, thus engaging a broader audience and increasing the volume and velocity of data reported. Opportunistically collected datasets, however, are characterized by spatial and temporal biases that pose both statistical and computer informatics challenges for the end user [74] and can limit their fitness of use for projects that require statistical confirmation of findings. For mosquito surveillance, where the distribution of larval habitats is associated nonrandomly with standing water, moveable containers, and ephemeral water features, an opportunistic data bias may be of less significance than in other research applications. However, it is also possible to use the GLOBE Observer app in investigations that require systematically sampled data [75], (see also Section 3.2, below).

The design of a citizen science project’s data information system significantly influences the quality of data collected. Information system components include the scope and activity of a project, data collection protocols, and the background and training of citizen scientists. For technology-mediated citizen science, the front-end human–computer interface, the data collection protocol, and instrumentation all contribute to the quality of reported data.

The GLOBE Observer data collection tasks were designed by scientists specifically for use by non-specialist volunteers and are both skill-appropriate and robust. Clear unequivocal data collection instructions, appropriate instrumentation, and data range and logic checks are foundational to GLOBE’s ability to deliver research-grade data [76].

The GLOBE Mosquito Habitat Mapper data information system includes many design features that contribute to consistency (data collection protocol), data accuracy (in-app tutorials, training workshops and webinars for volunteers), and data verification and validation (voucher photographs). Specific components of the data information system are summarized in Table 2, with examples of features that contribute to the information quality of Mosquito Habitat Mapper data. Additionally, back-end data management procedures, such as optical and automated photo validation, data range location, and logic checks, archiving, metadata availability, and data access procedures improve information quality [57].

### 2.6. Data Collection by Novices: Design Considerations

Both simple and complex data tasks are included in the Mosquito Habitat Mapper data collection system. The location, classification, photo-documentation, and mitigation of larval habitats are relatively straightforward tasks. However, the taxonomic identification of larvae is both time-consuming and challenging for novice users. In conjunction with each identification step in the in-app taxonomic key, citizen scientists have the option of selecting either “yes”, “no”, or “I’m not sure”. Citizen scientists are also able to submit photos without attempting specimen identification. These opt-out features of the app reduce false reports and enhance data quality [81]. The app’s information system collects user data on the number of steps undertaken by the citizen scientist while identifying their specimen and records the step at which an identification effort was terminated. These data provide a record of effort and precision in taxonomic identifications attempted by citizen scientists [77].

Despite careful design and deployment of the Mosquito Habitat Mapper, expert validation of citizen science submissions for some fields is required. For this reason, validated and curated datasets are made available periodically on the GLOBE Observer website. The most common errors observed in the data include the misidentification of specimens and user error when recording the geolocation of the habitat. These are described below.

### 2.7. Errors Associated with Citizen Scientist Larvae Identifications

The identification of larval taxa by non-specialists requires expert confirmation. To support current computer vision/A.I. research by scientists at the University of South Florida [82], photographs of larvae submitted by citizen scientists from Benin, Kenya, Senegal, and Madagascar were subjected to optical expert validation. *Anopheles stephensi* is an invasive species in Africa and is a competent vector for malaria parasites. Unlike the native *Anopheles* species, which prefer to oviposit in ponds, ditches, and swamps, *An. stephensi* also oviposits in containers [55]. The objective of the expert validation was to identify any *Anopheles* larval specimens reported from container breeding habitats and observe whether citizen scientists identified new areas of invasion. Voucher photographs of *Anopheles* associated with container habitats were tagged for further analysis.

While scanning for larvae photo candidates for computer vision analysis, we also characterized the accuracy of submitted citizen scientist identifications. For this study, we were only interested in whether citizen scientists were able to accurately identify *Anopheles.* larvae. *Anopheles* larvae have inconspicuous breathing siphons, whereas other taxa presented in the dichotomous key have elongated, conspicuous siphons. As the submitted voucher photos were reviewed, we tallied the number of accurate characterizations of elongated siphons (either not present or present), and whether specimens with inconspicuous siphons were identified correctly as *Anopheles*.

For each larval photo, we also noted if the specimen’s diagnostic characteristics were obscured, incomplete, or blurry. The outcomes of the manual expert validation of citizen scientist larvae identifications are presented in Table 3.

The manual expert validation process revealed common errors made by citizen scientists. The most frequent error made in identification was misidentifying a specimen as *Anopheles* when an elongated siphon was present. For most of these errors, the position of the larvae was obscuring or partially obscuring the siphon and would be difficult to discern without significant experience or expert knowledge.

A second common error was the misidentification of a pupal specimen as an *Anopheles* larva. Mosquito pupae are distinctive in shape and do not closely resemble the larval form, but like *Anopheles* larvae, they do not have a breathing siphon on their posterior end. We hypothesized that when these errors were made, the user only examined the posterior end. To reduce misidentifications, larva identification training resources will be revised to stress the importance of ensuring that a distinct head and thorax should be identifiable in *Anopheles* specimens, where no conspicuous siphon is observed.

Larval identification is a relatively challenging task that requires high-resolution photos and a careful examination of a specimen’s morphological features. In this sample, only 32% of larva photos were submitted with a citizen scientist’s identification. Of those, we correctly anticipated that the error rate in citizen scientist larval identification would be relatively high: over 60% of siphon identifications were incorrect.

Despite the low accuracy rate for identifying genera, the expert validation indicates that asking volunteers for their identifications is still very useful. The data show that there is a surprisingly low incidence (<1%) of misidentifying mosquito larvae as other aquatic organisms. This suggests that using the taxonomic key encourages citizen scientists to carefully examine their specimens and teaches them to recognize mosquito larvae with high accuracy.

In addition, providing the taxonomic key provides citizen scientists and community members with a tool to identify probable health threats in their community, so they can contact local or regional authorities with documented concerns. These two factors make it useful to include taxon identification in the app even if expert optical validation of identified larvae is ultimately required. An operational computer vision system for discerning the genus and species of larvae, currently in development, is expected to eliminate the need for manual optical validation [82].

### 2.8. User Errors Resulting in Inaccurate Geospatial Data Reporting

**Figure 7:**
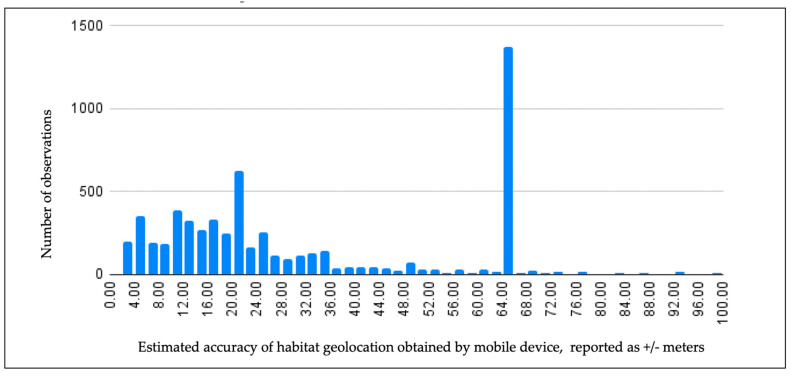
Estimated location accuracy of Mosquito Habitat Mapper habitat observations in 2021, automatically reported by the app as ± meters. (Filtered data, values < 100 m). The peak at ±64 m indicates user error.

A plot of locational data accompanying Mosquito Habitat Mapper observations for 2021 revealed an unexpected number of records reporting ±64 m estimated accuracy (Figure 7). The data anomaly reveals a common user error. Users are instructed to wait a minute and press the “refresh” button until an acceptable estimated accuracy is reported, between ±4–12 m, where possible. The spike in records with a reported estimated error of ±64 m identifies those users where they did not wait for a data return from the satellite. The data anomaly provides insight into a common user error associated with the data, and provides information that will allow us to fine-tune citizen science training and improve the geospatial accuracy of submitted observations.

### 2.9. Challenges Associated with Sampling Bias

Sampling bias is common in citizen science projects and is one of the most frequently cited data quality challenges [77,78]. Non-random sampling constrains the application of statistical tools, and is especially challenging for biodiversity projects where citizen scientists are contributing to a better understanding of the quantity and distribution of organisms in time and space. The common bias of observations clustering around where humans reside is another serious bias for these types of projects. In mosquito surveillance, however, the spatial bias of observations made in and around habitation centers is useful, because the observations and mitigation efforts support the health of these communities. While counts of mosquito larvae provide insight into the ubiquity of specific taxa, our primary research focus seeks to identify the kinds of mosquitoes associated with different larval habitat types [26] and how habitats are distributed with respect to land cover types [75]. These data have also been used to identify the presence of harmful invasive species in new contexts [82].

### 2.10. Data Interoperability

Environmental data are being generated at a volume, velocity, and variety otherwise impossible to achieve without public participation. However, because each citizen science project has unique goals, procedures, and protocols, there are limitations when efforts are made to combine data obtained from different projects for analysis. The Open Geospatial Consortium (OGC) [83] presents standards for geospatial data that promote findable, accessible, interoperable, and reusable (FAIR) data [84].

**Figure 8:**
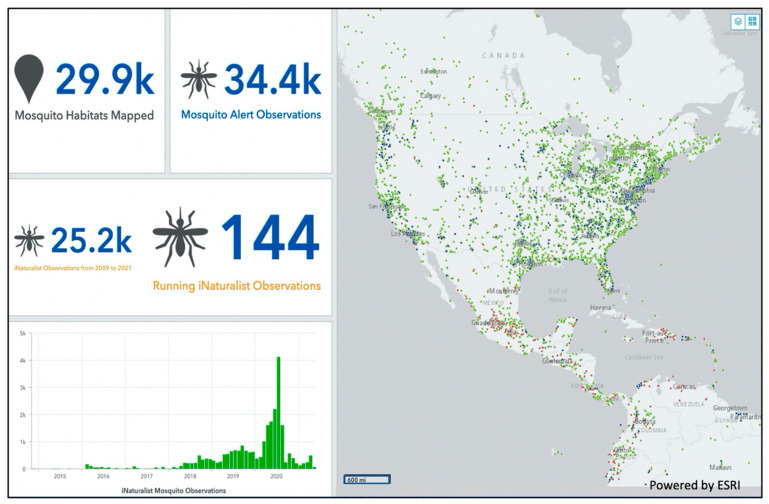
Screenshot of Citizen Science Cloud Dashboard demonstrating interoperability of mosquito data deriving from three independent citizen scientist projects: Mosquito Habitat Mapper, Mosquito Alert, and iNaturalist. Image Credit: Ryan Carney.

To increase the interoperability of GLOBE Observer datasets with other geospatial data, the GLOBE Mosquito Habitat Mapper and Land Cover datasets were aligned with the OGC data standard, The Sensor Things API [85]. The alignment procedure included the following steps: (1) contextualizing the questions GLOBE Observer datasets seek to answer, (2) detailing relevant measurement columns, and (3) describing data collection tools used [86]. The dashboard presented in Figure 8 demonstrates how the alignment of GLOBE Mosquito Habitat Mapper to OGC data standards enabled its interoperability with other citizen science datasets for research purposes. This work was conducted in partnership with The Global Earth Challenge (GEC) [87], a joint initiative with the Woodrow Wilson International Center for Scholars (The Wilson Center) and the US Department of State. The dashboard provides researchers with access to thousands of mosquito photos that are being used to develop computer vision software. 

## 3. Results

Low et al. presented GLOBE Mosquito Habitat Mapper data submitted by citizen scientists during 2017–2020 [26]. In the Mosquito Habitat Mapper’s first three years of use (29 May 2017–28 May 2020, over 24,000 data observations were reported. During that time, the number of observations steadily increased: from 2153 (2017), 6726 (2018), and 13,328 (2019), to a total of 24,983 by 28 May 2020). Some of the increase can be attributed the GLOBE Zika Education and Prevention project, which provided training and equipment to 22 countries in three GLOBE regions: Africa, Asia and the Pacific, and Latin America and the Caribbean [27]. An in-depth analysis of these data identified a pattern of replicate data submissions associated with training events and stimulated the development of data preprocessing tools now available for public use. Citizen scientists reporting data from three cities in Senegal identified *Anopheles* larvae in containers and suggested that citizen scientists potentially could play an important role in the identification of communities where the dangerous invasive malaria vector, *Anopheles stephensi*, is becoming established.

Citizen science data from Senegal also revealed an impressively high rate of source reduction. Mosquito Habitat Mappers from the cities of Dakar, Thilmakha, and Touba all reported source reduction action in over 90% of the larval habitats reported. This evidence demonstrated the role citizen science project participation can play in improving larval habitat mitigation locally.

As part of the GLOBE Zika Education and Prevention project, Aikpon et al. examined the indoors and outdoors population dynamics and biting behavior of *Ae. aegypti* in northern Benin [88]. The study was conducted over the course of one year to include wet and dry seasons. Six *Aedes* species were identified. While the seasonal biting behaviors were similar for both indoors and outdoors cases, the biting rate was higher outdoors during the afternoon hours than indoors. This was the first type of study conducted in Benin and has important implications for public health management of *Ae. aegypti* to limit the spread of arboviruses to humans.

### 3.1. Supporting Community Based Research: GLOBE Team Function and Geofencing Tool

**Figure 9:**
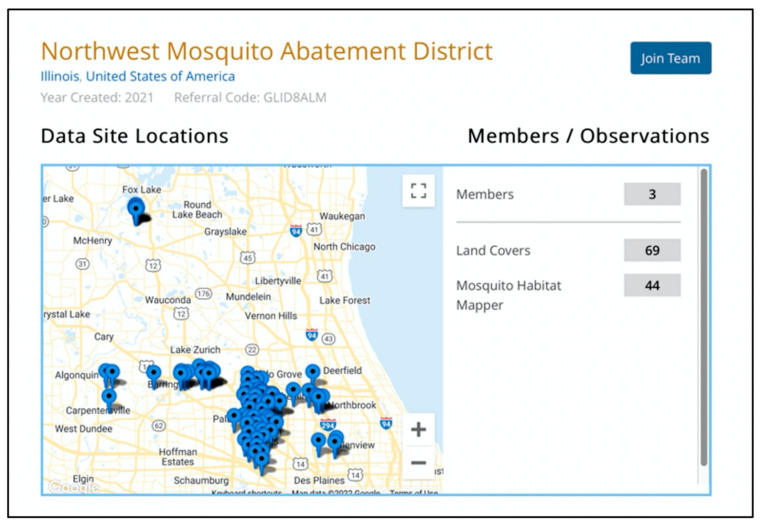
Example of autogenerated team map for Northwest Mosquito Abatement District, IL, USA. Source: The GLOBE Program.

Two built-in features of the GLOBE Observer app support project-level data collection, management and sharing. The first is the team tool. A user can join, create, or find a GLOBE team in the account settings. Any registered app user can create a team by entering a team name and country. Teams can be either open or private. The team function enables project leads to monitor observations of team members in an auto-generated map interface (Figure 9). Team data can be aggregated and accessed for analysis through the GLOBE ADAT using the built-in site filter.

The second is the new geofencing tool now being piloted for the GLOBE Observer app. The tool supports scientist or community requests for citizen science observations in a specific location. Data collection requests are submitted to the GLOBE Observer administrative team. The project is associated with a spatial bounding box on the app. Individuals who are using the app in an area with an active project are provided with an in-app prompt that there is an active project wanting data contributions. Two examples of projects under consideration for the new geofencing tool are presented below.

#### 3.1.1. Use Case 1: The Tire Removal, Education, Alteration, and Disposal (TREAD) Mosquito Management Project

The TREAD project is centered in Mobile and Baldwin Counties, Alabama, USA as well as Escambia and Santa Rosa Counties, Florida, USA. These are regions where several vector mosquito species either have been re-introduced or have newly invaded the region. Stacked, abandoned, and discarded tires are attractive oviposition sites for many species of mosquitoes [89]. Seasonal species composition would be good information to work toward and reduces the burden of tire removal and disposal.

The project tracks the seasonal species composition and mosquito oviposition behaviors in discarded tires. In doing so, TREAD seeks to promote public awareness of the health threat posed by discarded tires and to stimulate interest in mosquito surveillance by citizen scientists. A geofence in the app provides a push notification to citizen scientist app users within the research area of interest. Data collected in conjunction with the TREAD initiative are associated using the Mosquito Habitat Mapper’s team function, and citizen scientist recruitment is facilitated through the geofencing tool. In addition to the in-app notification, this project is supported by a data collection “Spare Tire Blitz” and social media posts [90].

#### 3.1.2. Use Case 2: Identifying Tree Hole Habitats in Residential Neighborhoods

The Northwest Mosquito Abatement District (NWMAD) is in the northwest suburbs of Chicago, Illinois, USA. In the US Midwest, *Ochlerotatus triseriatus,* the tree hole mosquito, is believed the primary vector of La Crosse Encephalitis (LAC) transmission to humans [91]. A total of five human cases of LAC have been reported in Cook County, Illinois in 2003–2021 [92].

To increase the surveillance of *Oc. triseriatus*, NWMAD personnel developed an applied study to map tree holes in forested and adjacent built-up residential areas. The dataset is captured using the Mosquito Habitat Mapper. NWMAD personnel mapped tree hole habitats in both protected nature preserves and adjacent residential areas. The Mosquito Habitat Mapper tool provide the opportunity for citizen scientists to assist in developing this dataset. Mass-planted silver maple trees lining the streets of these neighborhoods are particularly susceptible to the development of tree holes.

### 3.2. Use Case 3: GLOBE Observer Tool Supporting Agency Mosquito Control Operations

The development of predictive disease risk models requires an understanding of mosquito population dynamics in response to changes in meteorological and surface land cover conditions. Greenness, as described using the Normalized Difference Vegetation Index (NDVI), and structural land cover attributes are used to identify favorable conditions for mosquito growth and development. There is a pressing need for fine-grained, in situ field data that can be used to model the relationship between the presence of favorable breeding sites and land cover classes identified in satellite products [93,94]. This is especially important in regions where seasonal variability is pronounced, or where change is occurring rapidly. The GLOBE Observer mobile app supports the coincident collection of both Mosquito Habitat Mapper data and Land Cover data, and these data provide high-resolution land cover descriptions immediately surrounding oviposition sites. Together, they enable identification of microhabitat and microclimate features undetectable in Landsat (30 m) or Sentinel (10 m) image products [75]. By addressing this known data gap using both GLOBE Observer tools, citizen scientists have the potential to improve the fidelity of mosquito vector borne disease risk models that routinely employ environmental data obtained from satellites.

The GLOBE Observer mobile app and the GLOBE Data Information System streamline the collection and examination of coincident mosquito surveillance sampling sites and land cover, making these tools useful for NWMAD’s vector control program. NWMAD conducts mosquito and mosquito-borne disease surveillance and control operations within ~605.5 km^2^ (~233.8 mi^2^). This West Nile Virus (WNV) endemic area has reported 1278 cases and 65 deaths since the introduction of WNV in 2002 [95]. *Culex* spp., specifically *Culex restuans* and *Culex pipiens*, are the primary WNV vectors within NWMAD [96,97].

**Figure 10:**
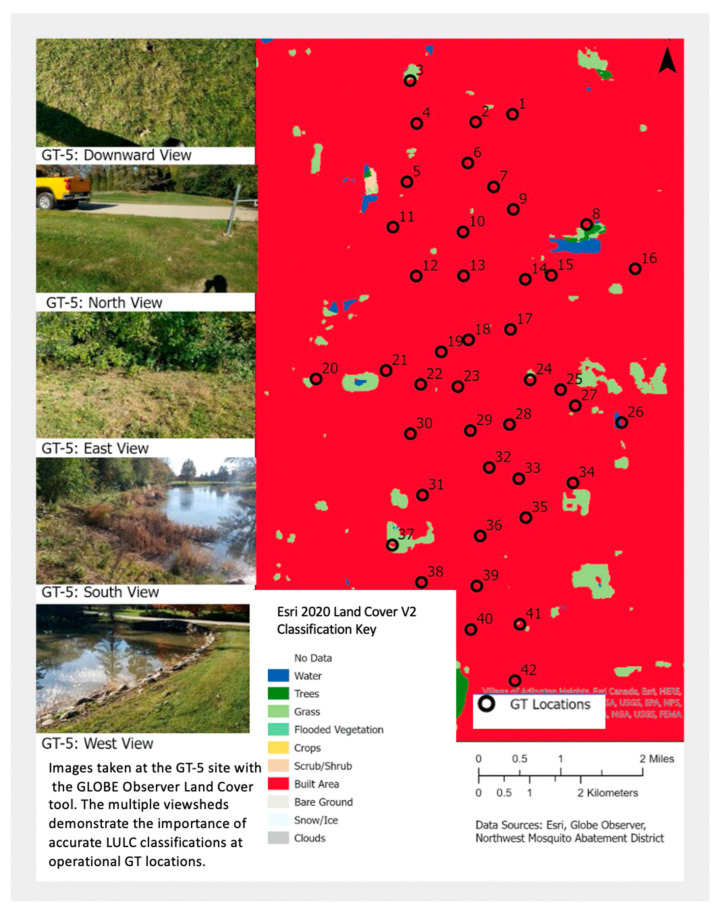
Position of 42 gravid traps superimposed over LULC classifications derived from European Space Agency (ESA) Sentinel-2 satellite imagery (10 m resolution). Base Map: Esri 2020 Global Land Cover. Left: Mosquito habitat features (water, trees, grass) documented using the GLOBE Observer Land Cover tool, but not identified in the land cover map product. Map from Northwest Mosquito Abatement District (NWMAD), northwest suburbs of Chicago, IL, USA. Source: Dan Bartlett, NWMAD.

To support targeted surveillance of *Cx. restuans/pipiens* through gravid trap (GT) surveillance, NWMAD began to investigate relationships between trap placement and land use land cover (LULC). While the preferred habitats of these mosquitoes are areas of human activity and urban development, it has been challenging to acquire LULC data that matched the fine spatial scale required for accurate description of 42 operational GT locations spaced over 43.62 km^2^. For example, the Environmental Systems Research Institute’s (ESRI’s) 2020 10 m Land Cover map has a reported overall accuracy of 86%. Nonetheless, it displays a “built area” classification for all trap locations [98]. However, many of the GT sites are located on grassland, within shrubs or trees, or directly adjacent to water bodies: mosquito habitats that are not identified in the map product. The GLOBE Observer Land Cover tool was used to capture photographs at each site to accurately document the LULC at each trap location. (Figure 10). In addition, the Mosquito Habitat Mapper tool was used to map above-ground mosquito sources near these locations.

NWMAD is also testing the usefulness of the two GLOBE Observer tools in surveillance of the eastern equine encephalitis (EEE) and LaCross Encephalitis (LAC) vector *Culiseta melanura.* NWMAD has partnered with regional researchers to record observations at potential *Cs. melanura* habitats. One challenge is identifying areas of suitable wetland habitat, such as sphagnum or tamarack. The goal is to use the GLOBE Land Cover tool to identify areas suitable for an independent classification of such habitats. To increase surveillance of LAC and *Oc. triseriatus*, NWMAD personnel have begun developing an applied study to map tree holes in forested and adjacent built-up urban areas.

**Figure 11:**
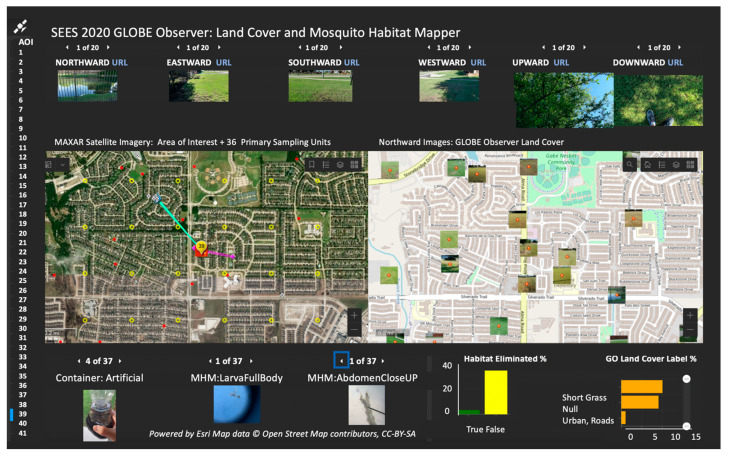
Data dashboard enabling rapid evaluation of land cover features and associated larvae (beta). Source: Peder Nelson.

The GLOBE Observer mobile app has value beyond the observations for mosquito control operations and LULC features in pursuit of accurate classifications. NWMAD is collaborating with the GLOBE Observer science team in the creation of a dashboard that allows viewing observation photographs across the region in a single online repository (Figure 11). Perhaps, most importantly, the application and tools can be used in public outreach to educate residents on the types of mosquito habitats that exist in their area, help them observe the landscape around them, and increase efficacy in mosquito surveillance, control, and research.

### 3.3. Application of the Mosquito Habitat Mapper to the Tracking of Invasive Species

An educational goal of the Mosquito Habitat Mapper is to raise public awareness of the contemporary impacts of climate change by directing attention to the sensitive response of mosquitoes to warming conditions. In the United States, the invasive vector mosquitoes *Ae. aegypti* and *Ae. albopictus* are increasing their geographical range poleward in response to warming conditions [2]. In addition, they are responding to changes in seasonality with longer breeding seasons. When the Mosquito Habitat Mapper app was developed, it was thought that citizen scientists could assist in documenting the range and seasonality trends for these vectors in the mid-latitudes. For this reason, the taxonomic key was designed to support citizen scientist identification of both *Ae. aegypti* and *Ae. albopictus.*

In response to the Zika epidemic in South America, IGES beta-tested the app with students and citizen scientists from underserved communities at high risk for Zika transmission. This work was conducted as part of USAID’s Combating Zika and Future Threats initiative [99], over two years (2017–2018), in six selected communities in Brazil and Peru. Field testing demonstrated that the older phones used by individuals in these communities precluded obtaining sufficient resolution photos to distinguish these two species from other *Aedes* taxa, but *Aedes* sp., *Culex* sp., and *Anopheles* sp. could be distinguished by the teacher and student citizen scientists with careful observation using an inexpensive 60–100× clip-on magnifier attached to the phone camera. Based on the data obtained during user testing, it was determined that asking citizen scientists to distinguish *Ae. aegypti* and *Ae. albopictus* from other *Aedes* species would be an unreasonable goal until the app could be paired with an AI-powered computer vision identification system.

*An. stephensi* is an invasive species recently introduced in Ethiopia, and as a competent vector for the pathogens that cause malaria in humans, it poses a new and serious health challenge in Africa [100]. Unlike the native African *Anopheles* mosquitoes *An. stephensi* is adapted to not only rural, but also urban environments. The *An. stephensi sensu stricto* urban type preferentially utilizes artificial water containers and storage tanks, as well as the range of cryptic habitats utilized by *Ae. aegypti* (such as evaporator coolers, cisterns, barrels, and roof gutters) [101]. Thus, citizen scientist reports of *Anopheles* specimens found in containers supports the identification and tracking of this dangerous invasive species in Africa. Control efforts will rely heavily on the early detection of this invasive species in new locations for rapid response. [102]. The Mosquito Habitat Mapper has been recognized as a potentially useful surveillance tool in the fight against *An. stephensi* range expansion [55] and is currently being used in Africa by citizen scientists and community entomologists as a surveillance tool to look for the presence of *An. stephensi*.

## 4. Discussion

### 4.1. GLOBE Mission Mosquito Campaign: Connecting Citizen Scientists with Training, Education, and Outreach Opportunities

A recent systematic literature review by Abourashed et al. identified some of the powerful reasons why citizen science programs that engage youth in disease surveillance can be effective from both a human health and a science literacy perspective [15]. They note that student participants have different motivations, scientific questions, and abilities than adults, and these need to be accommodated for youth audiences (Table 4). The following section describes the programmatic elements of Mosquito Habitat Mapper’s education and outreach strategy. 

To promote Earth system education as well as build and support a citizen science community of practice, the NASA Earth Science Education Collaborative sponsors the GLOBE Mission Mosquito campaign (2019–) [103]. The campaign addresses the well-known challenge of recruiting and retaining citizen scientist participants [104,105] by providing personally meaningful opportunities for volunteers to discover, participate, and contribute to the international challenge of mosquito vector surveillance and mitigation. This campaign is supported by a website, monthly webinars, occasional data challenges, educational assets, and public outreach via social media. An education advisory board includes international educators to assist in planning outreach efforts. Strategic collaborations with external organizations help broaden participation through formal education (e.g., curricula and lesson plans) and programs in out-of-school settings (e.g., libraries, scouts, camps, etc.).

**Figure 12:**
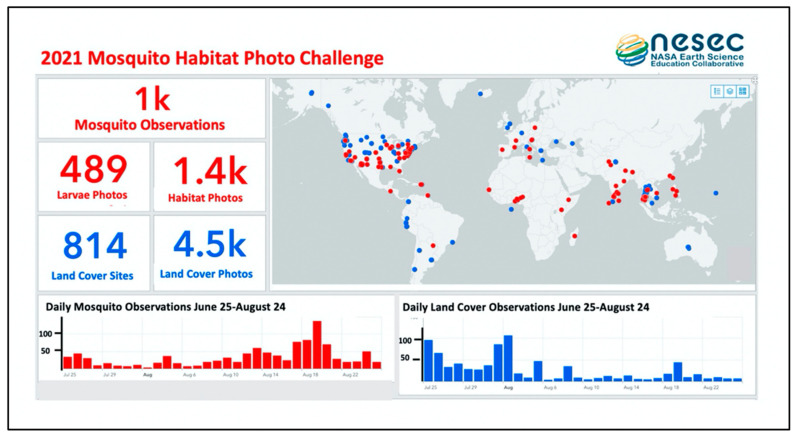
2021 Mosquito Habitat Photo Challenge Dashboard. Red dots indicate locations of Mosquito Habitat Mapper observations. Blue dots indicate locations of Land Cover observations. Source: Andrew Clark, IGES.

The primary mechanism for high-touch engagement is a monthly webinar series that includes technical training, reports of local project outcomes by international teams, sharing of education and outreach materials and activities, and informative discussions by scientists that highlight use cases of Mosquito Habitat Mapper data. In addition to the webinar series, the GLOBE Mission Mosquito campaign hosts periodic data challenges. Data challenges are designed to build excitement among citizen scientist participants, recruit new participants, and support the data needs of partnering scientific research projects. In 2021, the GLOBE Observer team hosted a month-long, Mosquito Habitat Photo Challenge, coordinating a request for detailed close-up larvae photos for computer vision/AI research [106]. Through NASA’s Established Program to Stimulate Competitive Research (EPSCoR), teams from the University of Vermont, University of Wyoming, New Mexico State University, and the University of Puerto Rico are using the land cover and mosquito habitat photos collecting during the challenge to support collaborative research through NASA Goddard’s AI Center for Excellence to develop automated image recognition systems. Data for these projects obtained during the Mosquito Habitat Photo Challenge are presented in Figure 12.

**Figure 13:**
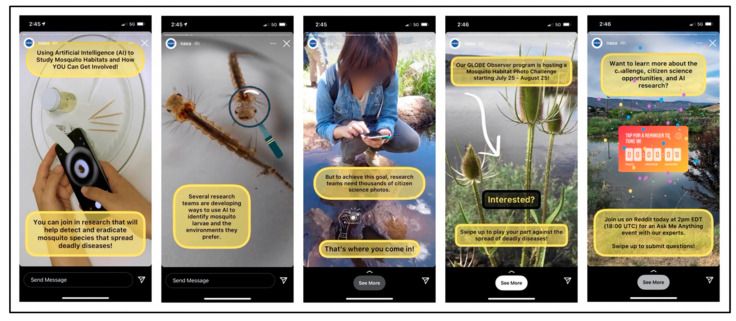
Scenes from the Instagram story about the 2021 Mosquito Habitat Photo Challenge. Source: GLOBE Communications Team.

Targeted social media campaigns accompany GLOBE Observer data challenges. Social media posts, scientist blogs, and events supporting the citizen scientist Mosquito Habitat Photo Challenge during 25 July–25 August 2021 reached over 2.5 million viewers through Facebook, Twitter, Instagram, YouTube, and Reddit. An Instagram and Facebook story reached 1.4 million viewers, with a completion rate of over 77% (Figure 13). The broad positive public recognition of NASA and having the opportunity to contribute to NASA science are strong motivation for GLOBE Observers [104]. Leveraging NASA social media boosts interest in and the adoption of the Mosquito Habitat Mapper app by citizen scientists.

### 4.2. Student Research

The NESEC team’s commitment to citizen science includes supporting students interested in using the data in original research projects. Each year, The GLOBE Program sponsors an International Virtual Science Symposium, where students submit research projects for evaluation by a team of international scientists. The GLOBE Mission Mosquito campaign provides pedagogic and technical support to teachers and secondary students to encourage the analysis of Mosquito Habitat Mapper data in student research projects. Cycles of classroom engagement with Mosquito Mapper by teachers are an unforeseen benefit of the educational programming: at a minimum, they promote episodic data collection in areas where otherwise surveillance would likely not take place.

Table 5 shows the reach of these activities using the number of submitted student research projects as a metric.

For the past three years, NESEC has led the Mosquito Mappers/Earth System Explorers summer research internship. This internship is a collaboration with the University of Texas, Austin/Texas Space Grant Consortium, which manages the NASA STEM Enhancement in the Earth Sciences (SEES) high-school research internship. In 2021, NASA scientist mentors supported 107 students in an 8-week virtual internship. The students collected data using the Mosquito Habitat Mapper and Land Cover tools and applied the data in an original team research project. Twenty-eight research projects were submitted to the GLOBE International Virtual Science Symposium and 23 posters were submitted to the secondary school student poster session at the 2021 American Geophysical Union (AGU) Fall Meeting.

An evaluation of the internship outcomes by Cho et al. demonstrated that citizen science tools and data could underpin a robust, cost-effective student research experience offered in a distributed virtual setting [107]. Mosquito Habitat Mapper, originally developed to support NASA educational objectives, lowers barriers to science participation online and broadens access to science, technology, engineering, and math enhancement opportunities.

### 4.3. Public Libraries: Hubs for Community Science

The role of the US public library has expanded dramatically in recent years, with libraries serving as a “cornerstone of community engagement and development” [108].

Public libraries are providing access not only to books but also access to computers, the Internet, field guides, subject matter experts, and equipment needed to participate in citizen science projects [109] and are emerging as important places to engage and share citizen science opportunities with the public.

**Figure 14:**
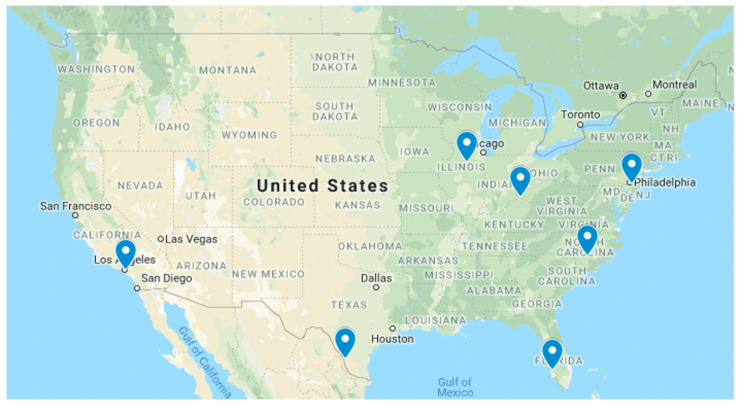
Locations of libraries field testing Mosquito Habitat Mapper resources, including California, Florida, Illinois, Kentucky, New Jersey, North Carolina, and Texas, USA. Source: Theresa Schwerin.

In 2018–2019, seven public libraries field-tested the GLOBE Observer Toolkit for Informal Educators in their programs (Figure 14). US libraries applied to field test the activities and were selected to include diversity in the geographic location, setting (urban, suburban, and rural), and type of programming (children, families, teens, adults, and seniors). The libraries completed 141 activity logs for programs that included 184 participants. The activity logs included details about the programs, how they used the resources, ease of preparation and use, and reflections on how engaging and effective the activities were. Overall library staff reported the activities were easy to prepare and set up, and the audiences were engaged. Specific suggestions provided input for revising and updating some of the activities to better meet informal educator and audience needs (e.g., simplify or clarify instructions, provide versions that were easier to print). The field test results also provided insights on adaptations for different programs and audiences.

The following are examples from two NESEC library partners that have integrated the Mosquito Habitat Mapper into their programs: Los Angeles Public Library (LAPL), CA, and La Salle Public Library, IL. These two libraries are markedly different in several ways. LAPL is a large, diverse library system with 73 branches; LaSalle Public Library is housed in a single building and serves an under-resourced community. Both libraries, however, share a commitment to engaging their community in STEM and citizen science.

#### 4.3.1. Los Angeles Public Library

The LAPL Neighborhood Science program implemented in 21 branches of Los Angeles Public Library is providing participants with knowledge of mosquitoes and tools to track, record, and share their habitat information with researchers and scientists using the Mosquito Habitat Mapper app. An important outcome is for program participants to know that they also have the power to stop the spreading of the mosquito population and prevent possible disease outbreaks through surveillance and mitigation using the GLOBE Observer app.

The Greater Los Angeles County Vector Control District now partners with the LAPL, and they collaborate on online mosquito programs and story times for families (in English and Spanish), culminating in an at-home field experience, using the Mosquito Habitat Mapper to check for nearby mosquito larvae habitats.

**Figure 15:**
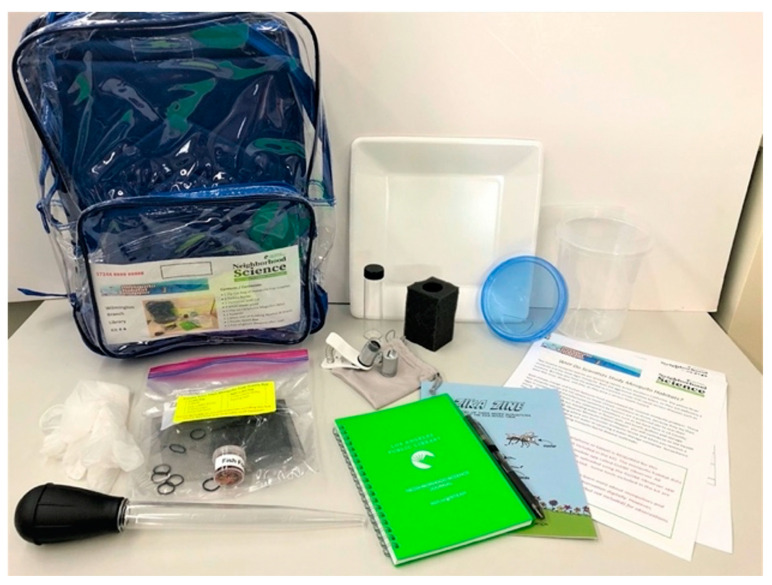
Example of a Mosquito Habitat Mapper DIY Citizen Science Kit. Source: LAPL.

The library has also developed Do It Yourself (DIY) Citizen Science Kits that incorporate resources from the Mosquito Habitat Mapper and can be checked out by patrons and teachers at local schools (Figure 15). The kit includes a clip-on macro lens, supplies for collecting larvae samples, Zika Zine comic (included in the GLOBE Observer Toolkit for Informal Educators), and instructions that include information developed with the GLOBE Mission Mosquito team explaining how mosquito-borne disease is relevant to Southern California. In addition, LAPL offers online mosquito programs and story times for families (English and Spanish) that culminate in at-home field experiences, using the Mosquito Habitat Mapper to check for nearby mosquito larvae habitats.

#### 4.3.2. LaSalle Public Library, Illinois

**Figure 16:**
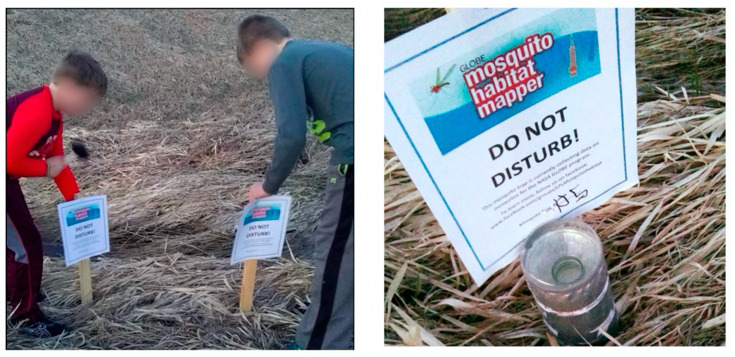
LaSalle Public Library Mosquito Mappers post “do not disturb” signs near larvae traps. The signs include the LaSalle Public Library Facebook Group for the project, providing additional outreach and awareness. Source: LaSalle Public Library.

The library has hosted Mosquito Habitat Mapper programs in a nearby nature preserve where participants set and examined traps, still-water areas, and streams (Figure 16). On neighborhood walks, participants identified unwanted mosquito habitats throughout the community. Because LaSalle is a river town, many of these unwanted habitats are found in debris along the Illinois River. The library programs place special emphasis on litter clean-up to remove unnatural habitats. These outdoor activities are paired with indoor programs that teach participants about the life cycle of mosquitoes, their appearance at each stage, the diseases related to mosquitoes as vectors, both locally and around the world.

### 4.4. Educational Materials

**Figure 17:**
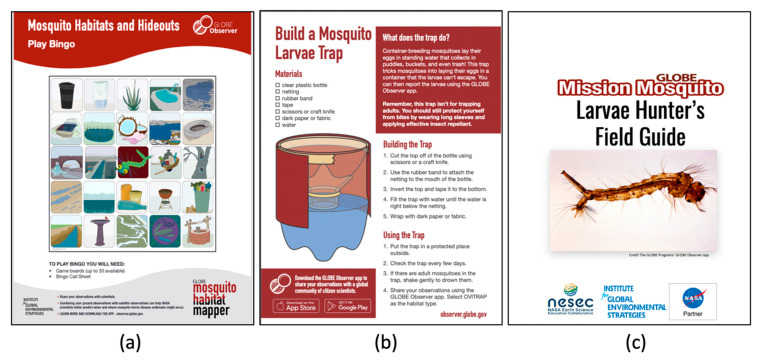
Mosquito Habitat Mapper Activities and Games. (**a**) Mosquito Habitats and Hideouts Bingo, (**b**) Build a Mosquito Larvae Trap, and (**c**) The GLOBE Mission Mosquito Larvae Hunter’s Field Guide. These activities reinforce concepts and allow practicing skills needed for taking observations, e.g., identifying different habitats where mosquitoes can breed, understanding habitat characteristics that meet mosquito needs, and practice identifying mosquito larvae from specimen photos. Source: IGES.

A wide range of educational materials supporting use of the Mosquito Habitat Mapper are available at no cost to the public (Figure 17). In addition to hands-on activities, games, and lessons, the GLOBE Mission Mosquito campaign website also provides access to instructional videos, a library of informational webinars, and a citizen science blog. Together, these resources reinforce the science knowledge and science skills needed for taking Mosquito Habitat Mapper observations.

Because citizen science offers a unique opportunity for learners to participate in authentic, inquiry-based science, data collection activities using the Mosquito Habitat Mapper have been incorporated into curricula for use in secondary school classrooms (US middle and high school) that were developed by collaborating organizations. These include the *Mosquito! Community Research Guide* [110], a multi-module curriculum developed by the Smithsonian Science Education Center and the *Vector-borne Disease Module* created by the National Institutes for Health, National Institute of Environmental Health Science [111].

#### Toolkit for Informal Educators

GLOBE Mission Mosquito activities that can be used in out-of-school programs are also available as part of the GLOBE Observer Toolkit for Informal Educators [112]. The toolkit has been used in various informal education settings, including field testing with public libraries and with troops of Girl Scouts of the United States of America working on Journey awards. A *Girl Scout Journey* is an extended engagement with a topic that culminates in a *Take Action* project to address a community concern. The Mosquito Habitat Mapper is one of a small number of curated projects available on SciStarter for the Girl Scouts USA *Think Like a Citizen Scientist Journey* [113]. The GLOBE Observer team developed a Girl Scout Facilitators Guide for troop leaders that is available in the GLOBE Observer Toolkit. The guide provides support to troop leaders who may not have a strong science background [114].

### 4.5. Role of Outreach in Data Collection

**Figure 18:**
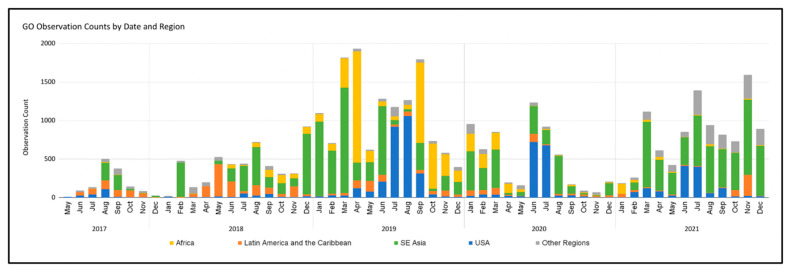
Mosquito Habitat Mapper Observations by identified by month, year, and region.

Although the Mosquito Habitat Mapper welcomes the participation of all citizen scientists anywhere and anytime, the impact of targeted outreach events, including data campaigns and scientist-led research initiatives, is observed in the data upload statistics (Figure 18). Pilot testing of the tool with teachers and schools in Brazil and Peru account for the peak participation of Latin American countries in March–June 2017 and 2018. GLOBE’s Zika Education and Prevention Project supported Mosquito Habitat Mapper workshops in Africa, Latin America and the Caribbean, and Southeast Asia and the Pacific, and increased African participation from mid-2018–mid-2020. High school research interns participating in the SEES Earth System Explorers and Mosquito Mappers teams were predominantly responsible for the spike in US data uploads in June–July 2019–2021. Peak numbers of data uploads coincided with the Mosquito Data Blitz in April 2019. The strength of an active and ongoing student outreach program in Thailand is observed in the consistently high number of data uploads from southeast Asia. To date, education and outreach programs, events, and research projects drive data collection using the Mosquito Habitat Mapper. The connection with schools and community organizations fosters data collection and reporting, especially when mosquito-borne disease risk is at background levels and not perceived as an urgent health threat requiring personal action by the public.

## 5. Conclusions

GLOBE Observer is a citizen science mobile app paired with an open data infrastructure. The app’s Mosquito Habitat Mapper tool enables the collection of time-stamped geospatial data with photos of larval habitats and larval specimens. The Land Cover tool captures the associated environmental context of the larval habitat, including LULC statistics and vegetation health. The diverse data obtained using these tools can support a wide variety of research projects, as well as local participatory community-based surveillance campaigns.

Available at no cost to the user in 126 countries in 14 languages, the GLOBE Mosquito Habitat Mapper serves as a global public good [115]. As with many public goods, the future benefits of an international mosquito surveillance database are still unknown. The usefulness of Mosquito Habitat Mapper data in *An. stephensi* surveillance [55,82] is an outcome unforeseen when the app was initially developed.

One thing we have learned from the COVID-19 pandemic is how important it is to pivot quickly in response to a global health threat [116]. The planned capability for GLOBE Observer to push messages to citizen science users in specific geographic locations readies the Mosquito Habitat Mapper tool for potential rapid deployment during a future mosquito-borne disease epidemic, even in low-resource communities where no other mosquito surveillance tools are readily available.

GLOBE Observer is a multilingual app that works on and offline on a variety of mobile platforms, including older mobile devices. By providing open access to all educational assets, an easy-to-use app, and a persistent, stable data archiving solution, we hope to reduce some of the barriers that prevent the development of local or regional scale vector surveillance and mitigation initiatives. However, we recognize that there are still barriers to adoption of the Mosquito Habitat Mapper in many parts of the world. There are more than 6 billion smartphones in use by approximately 83% of the world’s population [117], but the digital divide continues to disenfranchise populations who need tools, such as the Mosquito Habitat Mapper the most, where mosquito-borne diseases are endemic and municipal mosquito control is underfunded or nonexistent.

Through the expansion of our network, and partnering with schools, community organizations, and mosquito control programs, we hope to foster the robust reporting and archiving of data collected in geographic regions that are otherwise underexamined and underreported. In this way, the Mosquito Habitat Mapper stands as a powerful tool to crowdsource fine spatial scale mosquito ecology data, monitor, and mitigate the spread of vector mosquitoes, and empower citizen scientists to improve local heath and reduce the risk of mosquito-borne disease in their communities.

## Figures and Tables

**Table 1:** List of representative citizen science mosquito surveillance projects, 2012–present.

Project	Report Method	Data Type	Life Cycle Stage	Surveillance Target	Participant Focus	Date	Geographic SCALE	Country of Origin
Abuzz [19]	Web	Audio	Adult	Wingbeat species ID	Public	2017–2018	Country	USA
Atrapar el Tigre [20]	App	Photo	Adult	*Ae. albopictus*	Public Students	2013–2014	Country	Spain
Sem Dengue–BreakZika [21]	App	Photo Text	Larva	Larva habitat symptoms	Public	2016–2017	Country	Brazil
Caza Mosquitoes [22]	App	Photo	Adult	Mosquito	Public	2017–	Country	Argentina
Citizen AcTS [23]	In situ expert ID	Specimen	Adult	*Ae. albopictus*	Field expert Public	2016–2017	Local (one city)	USA
Dengue Chat [24]	Web Social media	Photo	Larva	Larva habitat, disease case	Public	2015–	International	Nicaragua, Mexico, Brazil, Paraguay Colombia
GO Mosquito Community Challenge [25]	App	Photo	Larva	*Ae. aegypti, Ae. albopictus* ID, counts, larval habitats, source reduction	Students	2017–2019	Local (6 cities)	Brazil, Peru
GLOBE Observer Mosquito Habitat Mapper [26]	App	Photo	Larva	(See above)	Students Public	2017–	International (126 countries)	USA
GLOBE Zika Education and Prevention Project [27]	App	Photo	Larva	(See above)	Students Public	2018–2021	International (22 countries)	Africa, Asia, and Pacific, Latin America, Caribbean
Great Arizona Mosquito Hunt [28]	Mail	Egg paper	Egg	*Aedes* sp.	Students	2015–2017	Regional (State: AZ)	USA
Humbug [29]	App	Audio	Adult	Wingbeat species ID	Public	2014–	International	UK
Kidenga [30]	App	Text	Adult	Adult activity, disease cases	Public	2016–	Regional (SW)	USA
iMoustique [31]	App	Photo	Adult	*Ae. albopictus*	Public	2013	Country	France
iNaturalist: Mosquito AI [32]	App	Photo	Adult	recent invasive species	Public	2021–2022	Regional	USA
iNaturalist: Mosquitoes in HI [33]	App	Photo	Adult Larva	invasive species	Public Students	2015–	Regional (State: HI)	USA
Invasive Mosquito Project [34,35]	Mail	Egg paper	Adult	*Ae. aegypti,* *Ae. albopictus*	Students Public	2016–	Country	USA, Canada
Lansanka Model [36]	Paper Web	Text	Larva	Larva habitats	Public	2014–2015	Local	Thailand
Mo-Buzz [37]	App	Photo	Adult Larva	Larva habitat, bites, symptoms	Public	2013–	Local	Sri Lanka
Mosquito Alert [38]	App	Photo	Adult Larva	Invasive *Aedes* sp., larval habitats, bites	Public	2014–	International	Spain
MOSapp/DI Sapp [39]	App	Text	Adult	Vector mosquitoes disease cases	Public	2015–	Country	India
Mosquito Census [40]	Web Mail	Specimen	Adult	All mosquitoes	Public	(2019)	Country	New Zealand
Mosquito Mapper [41]	App	Photo	Adult	All mosquitoes	Public	2017	City (Berlin)	Germany
Mosquito Reporting Scheme/ Mosquito Watch [42]	Mail	Specimen	Adult	Mosquitoes	Public	2005–2012	Country	UK
Mosquito Stoppers [43]	Web	Text	Adult	Nuisance mosquitoes	Public	2014–2015	City (Baltimore)	USA
MosquitoWEB [44]	Mail	Specimen	Adult	All species	Public	2014–	Country	Portugal
Mozzie Monitors [45]	Web	Photo	Adult	Gravid trap specimens	Public	2018–2019	Regional	Australia
Mueckenatlas [46]	Mail	Specimen	Adult	All species	Public	2011– open	Country	Germany
Muggenradar [44]	Web, mail	Specimen	Adult	Nuisance mosquitoes	Public	2014–2015	Country	Germany
North American Mosquito Project [47]	Mail	Specimen	Adult	All mosquitos from trap	Public	2011–2015	International	USA, Canada
TopaDengue [48]	App	Photo	Larva Pupa	Larva habitat monitoring, *Ae. aegypti*	Students Public	2018–2019	Local	Paraguay
West Nile Virus Vector Project [49]	Mail	Specimen	Adult	*Ae. albopictus*	Public		Country	Netherlands
ZanzaMapp [50]	App	Text	Adult	Nuisance mosquitoes	Public	2016–2018	Country	Italy
Zika Mozzie Seeker [51]	Mail	Egg paper	Eggs	*Ae. aegypti, Ae. albopictus*	Public	2017–	Regional	Australia
Unnamed project [52]	Paper tablet	Map	Larva	Anopheles larva habitat	Public	2012–2013	Local (3 villages)	Tanzania
Unnamed project [53]	Mail	Egg	Egg	Invasive *Aedes* sp.	Public	2017	Regional (6 provinces)	Austria
Unnamed project [11]	Paper	Specimen	Adult	Nuisance mosquitoes	Public	2017–2018	Local (12 villages)	Rwanda

**Table 2:** Description of GLOBE Mosquito Habitat Mapper Information System related to data quality. Categories based on data collection scenarios described by Lukyanenko et al. [77] and data quality considerations and processes discussed in Kosmala et al. [78], Lewandowski and Specht [79], and Wiggins et al. [80].

Information System Components	Subdimension	Characteristics	Example from Mosquito Habitat Mapper
Scope and activity	Geographic scale	Large, unbounded	Anytime, anywhere in 126 GLOBE countries
	Task type	Easy–hard	Volunteers can choose tasks they want to perform and have time to perform, making participation available to citizen scientists with a wide variety of skill levels and interests. Easy = photographing standing water source, dumping water. Hard = taxonomic identification of a specimen
	Data collection tasks	Specified, closed	Standardized protocol, categorical variables, collection conditions reported
	Public participation model	Collaborative technology-supported	Volunteers encouraged to collect, analyze, interpret, and disseminate outcomes
Citizen scientists	Recruitment	Inside and outside GLOBE network	GLOBE training events, social media, publicly advertised data challenges, teacher professional development workshops and webinars
	Ability level	Experts/nonexperts in project domain	Anyone aged 13+ years can participate
	Training	Minimal training required	Initial: in-app tutorial, instructional video, and eTraining module Ongoing: GLOBE Mission Mosquito Campaign webinars, workshops, and events
	Volunteer Assessment	Optional certification via eTraining test	GLOBE eTraining module and certification quiz
Device collection features	Geospatial data Collection	Automated, contributor-centric	Geospatial data are obtained automatically in-app. Users must wait until the satellite fix returns position with acceptable accuracy, and click on the “reset” button at 30 second time intervals until accuracy of ±12 m is achieved
	Taxonomic identification	Instance/ attribute-based	(1) Mosquito taxonomic attributes (such as siphon) identified individually prior to assigning to class (taxon) (2) Choice option: “I’m not sure” to reduce false identifications
Raw data	Data quality management	Contributor-centric	Robust protocols and training support citizen scientist skill development and minimize errors in data reporting
	Data documentation	Metadata	GLOBE Data User Guide
	Access	Open data	GLOBE Visualization System, ADAT, API, Earth System Explorer Portal
	Data analysis	Significant and advanced data cleaning and post-processing required	Characteristic of non-expert data collection system
Preprocessing support and quality assurance procedures	Database range and logic checks	Algorithmic identification of outliers	GLOBE Data Information System
	Expert validation	Data review	Photo approval system, expert validation, AI (in development) currently expert-validated cases available in curated datasets
	Tracking volunteer performance	Origin of each record identified	Each citizen scientist is identified anonymously, enabling quality tracking
	Access to processing algorithms	Support for reusable and reproducible data processing steps	Jupyter Notebooks (GLOBE data) Earth System Explorer data portal (beta) Streamlite
	Voucher photographs	Mosquito habitats and larval specimens	Manual and AI photo approval system rejects inappropriate photos
Data users	Research Topics	Known (satellite data interpretation) and unknown (evolving)	Earth system science, predictive models of vector disease, environmental justice action, operational vector control management, satellite data interpretation, computer vision research (AI), invasive species monitoring
Education outreach	Pedagogic assets and programs	Student research applications	GLOBE US Student Research Symposium GLOBE International Science Symposium

**Table 3:** Outcomes of expert validation of citizen scientist identifications of larval specimens found in containers, submitted from Benin, Kenya, Senegal, and Madagascar.

Expert Validation of Citizen Scientist Identifications	Benin	Kenya	Senegal	Madagascar
**Correct Identifications**				
Not *Anopheles*: Present siphon identified correctly	8	38	192	4
*Anopheles*: Absent siphon identified correctly	4	2	23	1
**Incorrect Identifications**				
Siphon identified as present, but is absent	1		65	
Siphon described as absent, but is present	3		236	
Pupa misidentified as Anopheles larva	2		22	
Identified as *Anopheles*, but photo quality is insufficient to confirm (blurry, bad angle, etc.)	2		93	
Specimen is misidentified as a mosquito larva	2		1	
Total identified specimens from containers/total	22/233	40/67	632/2211	5/6
% Accuracy based on voucher photos	55%	100%	34%	100%
% Specimens with attempted identification	9%	60%	29%	91%

**Table 4:** (a) Strategies to Implement Effective School CS Programs in Infectious Disease Surveillance, as outlined by Abourashed et al. [15]. (b) Alignment of Mosquito Habitat Mapper’s programmatic attributes to each strategy.

	Effective Strategies for School-Based Citizen Science Programs	Examples from Mosquito Habitat Mapper Programming
(1)	Consider program participants: student participants are different from adults in a citizen science program. Developing programs that account for student motivation, scientific curiosity, and capabilities are crucial for a program’s success.	Student motivations: desire to conduct locally meaningful work; contribute to community health; earn recognition from other students, teachers, and community; and obtain data for science fair projects. Fulfills service learning and environmental justice project requirements in some district curricula.
(2)	Support current school curriculum and initiatives: citizen science projects that align well with teachers’ lesson plans and standards make implementing a citizen science project less demanding.	Mosquito Habitat Mapper educational resources are designed as activities that support educational objectives of US Next Generation Science Standards.
(3)	Create simple and clear protocols: students focus on following procedures. Protocols should be explained plainly and be easy to follow. In addition, data collection should be accessible.	The Mosquito Habitat Mapper app tool is easy to use and students can participate in those data collection tasks that are appropriate to their interest and skill levels. Beta tested with youth (13–17 years old).
(4)	Take advantage of appropriate technology: using technology that is portable, such as smartphones, can aid implementation of citizen science projects. This also supports rapid data collection.	The GLOBE Observer mobile app work on older devices and offline. Currently available in 14 languages.
(5)	Maintain open communities and feedback with students and teachers: students should understand their role as citizen scientists. Students and teachers need to know why they are collecting data and why they are doing so in a specific way. Discussing the impact of their work and how long the data will be available for analysis is also valuable.	The app’s *team* function facilitates teacher tracking of student data. Monthly webinars are used to maintain a community of practice among participating educators and students. The webinars also connect citizen scientists with professional scientists who are using the data in their research.
(6)	Promote community outreach: citizen science is a community-driven scientific initiative. Involving students and their community members enhances scientific confidence and strengthens civic cooperation.	GLOBE Observer connects with both adult and student audiences through libraries, which serve as community science hubs throughout the US, especially in rural and underserved communities. Community partners identify their own data collection and participation goals.
(7)	Spread knowledge gained through experience and results: publicizing citizen science projects and showing collaborations between experts and non-experts can build the public’s trust in science and combat scientific misinformation.	Coordinated social media campaign, webinars connecting citizen scientists with experts. NASA actively encourages scientific publication with citizen scientists as co-authors (including youth).
	(a)	(b)

**Table 5:** The number of GLOBE International Virtual Science Symposium projects submitted by students using Mosquito Habitat Mapper data. The numbers for 2022 are preliminary and the number of total submissions was not available (n/a) at time of publication. Data source: The GLOBE Program.

Grade Level	2022	2021	2020	2019	2018
Upper secondary (grades 9–12 US)	44	58	26	9	6
Lower secondary (grades 5–8 US)	3	7	12	8	2
Upper elementary (grades 3–4 US)	0	1	5	1	0
Total submissions, all topics	220	242	265	238	113
% IVSS Projects analyzing Mosquito Habitat Mapper data	21%	27%	16%	8%	7%

## Data Availability

GLOBE Observer data are publicly available at https://www.globe.gov/globe-data (accessed on 30 March 2022). Raw data analyzed in this project were accessed from this location. The Python code to read, analyze, and visualize GLOBE data for this article, as well as the analyzed data sets, are available on https://github.com/IGES-Geospatial/GLOBE-Data (accessed on 30 March 2022). Dashboard access to Mosquito Habitat Mapper and Land Cover data is available at https://geospatial.strategies.org/ (accessed on 30 March 2022). To best enable re-use with attribution, all data products from the Earth System Exploration Portal are licensed with a Creative Commons Attribution 2.0 Generic (CC BY 2.0) data license.
